# Semantic segmentation of HeLa cells: An objective comparison between one traditional algorithm and four deep-learning architectures

**DOI:** 10.1371/journal.pone.0230605

**Published:** 2020-10-02

**Authors:** Cefa Karabağ, Martin L. Jones, Christopher J. Peddie, Anne E. Weston, Lucy M. Collinson, Constantino Carlos Reyes-Aldasoro

**Affiliations:** 1 Research Centre for Biomedical Engineering School of Mathematics, Computer Science and Engineering, Department of Electrical & Electronic Engineering, City, University of London, London, United Kingdom; 2 Electron Microscopy Science Technology Platform, The Francis Crick Institute, London, United Kingdom; 3 giCentre, Department of Computer Science, School of Mathematics, Computer Science and Engineering, City, University of London, London, United Kingdom; Beijing University of Technology, CHINA

## Abstract

The quantitative study of cell morphology is of great importance as the structure and condition of cells and their structures can be related to conditions of health or disease. The first step towards that, is the accurate segmentation of cell structures. In this work, we compare five approaches, one traditional and four deep-learning, for the semantic segmentation of the nuclear envelope of cervical cancer cells commonly known as *HeLa* cells. Images of a HeLa cancer cell were semantically segmented with one traditional image-processing algorithm and four three deep learning architectures: VGG16, ResNet18, Inception-ResNet-v2, and U-Net. Three hundred slices, each 2000 × 2000 pixels, of a HeLa Cell were acquired with Serial Block Face Scanning Electron Microscopy. The first three deep learning architectures were pre-trained with ImageNet and then fine-tuned with transfer learning. The U-Net architecture was trained from scratch with 36, 000 training images and labels of size 128 × 128. The image-processing algorithm followed a pipeline of several traditional steps like edge detection, dilation and morphological operators. The algorithms were compared by measuring pixel-based segmentation accuracy and Jaccard index against a labelled ground truth. The results indicated a superior performance of the traditional algorithm (Accuracy = 99%, Jaccard = 93%) over the deep learning architectures: VGG16 (93%, 90%), ResNet18 (94%, 88%), Inception-ResNet-v2 (94%, 89%), and U-Net (92%, 56%).

## 1 Introduction

The study of cells and their organelles has interested scientists from the early days of Hooke and van Leeuwenhoek to the formulation of cell theory by Schleiden and Schwann [1]. Since then, presence or absence of cells, shapes, inner components, interactions, regulation of processes, environment, and many other characteristics have been thoroughly analysed, especially trying to relate these to conditions of health and disease [2–7]. To observe cells, it is necessary to use microscopy and one of its many different techniques like light, fluorescence or differential interference microscopy. Electron Microscopy (EM) can provide resolving power several orders of magnitude higher than conventional light and fluorescence microscopes and thus it is ideal to observe small structures of the cellular environment. Modern EM instruments allow the acquisition of contiguous images of a sample by slicing very thin sections from the top face of the resin-embedded sample with an ultramicrotome diamond knife [8]. Once the sample is sliced, the slice itself is discarded, the sample is raised into the imaging position and the scanning process continues for a given number of slices, thus creating a three-dimensional data set of contiguous images. This process is called *Serial blockface scanning EM* (SBF SEM) [9].

The nuclear envelope (NE) is a bi-layer membrane that separates the nucleus with the chromosomes from the rest of the cellular compartments [10] and contains a large number of membrane proteins with sophisticated roles and functions [11–14]. The structure and condition of the NE is of huge importance as it has been related to viral infections [15–19], Muscular dystrophy [20], Cancer [21–25], Osteoporosis [26], Cardiovascular diseases [27–29], other diseases [30–32], and ageing [33–35]. Therefore, algorithms for the segmentation, visualisation and analysis of the NE could provide parameters to understand the conditions of health and disease of a cell.

The segmentation of cells and their environment through image-processing tasks have been important for many years and numerous algorithms have been proposed. Candia *et al*. summarised the importance of objective analysis emphatically in the following way: “we need unbiased, mathematically robust, scalable methods that allow us to identify key parameters that consistently characterise cell subpopulations … to build signatures of health and disease” [3]. PubMed [36] contains more than 33,000 entries with the words cell and classification or segmentation in the title and abstract ((classification[Title/Abstract] OR segmentation[Title/Abstract]) AND cell[Title/Abstract]). The number of entries drops considerably to less than 1000 when the keyword “electron” is added to the search. Segmentation of images acquired with electron microscopy (EM) is difficult for several reasons. The considerable increase of size and resolution as compared with light and fluorescence microscopy provides complex morphological structures. Whilst fluorescence microscopy allows several channels that identify structures of interest, EM only provides a grey scale image and with a reduced contrast between the structures of interest and the background. Furthermore, when serial sections are obtained, the images are transformed into a volumetric data set.

Recently, advances in machine learning and artificial intelligence, especially those related to deep learning architectures [37], have revolutionised image processing tasks [38–43]. Several deep learning architectures [44–46] have obtained outstanding results in difficult tasks such as those of the ImageNet Large Scale Visual Recognition Challenge (ILSVRC) [47]. Not surprisingly, deep learning has become a popular tool for segmentation. Convolution neural networks (CNN) [48], are versatile and have been shown to be very effective for a wide range of tasks including traditional tasks like object detection [49, 50] image classification [51–55] and segmentation [56], to more specific ones like quantification of heart ventricles [57]. The U-Net architecture, proposed by Ronneberger [58] has become a widely used tool for segmentation and analysis. It recently became the most cited paper presented in the prestigious MICCAI conference. Cireşan [59]*et al*. applied deep learning architectures to detect membrane neuronal and mitosis detection in breast cancer [60]. Within EM studies, deep learning has been applied to analyse mitochondria [61, 62], synapses [63] and proteins [64].

Deep learning architectures have two main limitations: 1) they require a large amount of training data and 2) they require significant computational power. As graphics processing units (GPUs) become more popular, the main limitation is thus the scarcity of training data [52, 65–69].

In this work, four deep learning architectures, VGG16 [46], ResNet18 [70], and Inception-ResNet-v2 [71], and U-Net [58] were used to perform the semantic segmentation of HeLa cells. VGG16 has been widely used in a variety of image segmentation problems. ResNet solves the problem of vanishing/exploding gradients and was the winner of ILSVRC 2015 [47]. Inception-ResNet-v2 employs dropout to avoid overfitting and is seen as the successor of GoogLeNet [71]. The U-Net architecture contains two paths, first one path that contracts by reducing the size of the input images through which the context is capture, and a second expanding path; symmetric to the first, through which precise localisation is obtained.

These networks were selected because of their good balance between accuracy and computational complexity, especially ResNet and Inception-ResNet-v2, which outperform other common configurations and are at the Pareto frontier considering accuracy and complexity [72–74].

The first three algorithms were pre-trained with ImageNet and then fine-tuned with training data prepared for this work. These were then compared with a traditional image processing algorithm [75]. The U-Net was trained from scratch with a series of training images and labels constructed from a subset of the data and the ground truth. The image processing algorithm followed a pipeline of traditional tasks: low-pass filtering, edge detection, dilation, generation of super-pixels, distance transforms, mathematical morphology, and post-processing to segment automatically the nuclear envelope and background of HeLa cells. Previously, the image processing algorithm was compared against active contours (snakes) and it outperformed active contours in both accuracy and time [75]. Improvements and refinements of the snake model (e.g. [76]) keep the snakes as an active research topic in computer vision and image analysis.

The main contributions of this work are: (a) The objective comparison of five semantic segmentation strategies, one traditional image processing and four deep learning. (b) These strategies were compared through the semantic segmentation of the nucleus, nuclear envelope, cell and background of three hundred slices of a HeLa cell observed with electron microscopy. (c) Open source code for all the segmentation strategies, which has been made available through GitHub. All the programming was performed in Matlab^®^ (The Mathworks^™^, Natick, USA). (d) The four-class ground truth for 300 slices has been created and made available through Zenodo. The EM data is available through EMPIAR (see S1 Code).

## 2 Materials and methods

### 2.1 HeLa cells preparation and acquisition

Details of the cell preparation have been published previously [77], but briefly, the data set consisted of EM images of HeLa cells, which were prepared and embedded in Durcupan resin following the method of the National Centre for Microscopy and Imaging Research (NCMIR)[78].

### 2.2 Image acquisition

**Fig 1:**
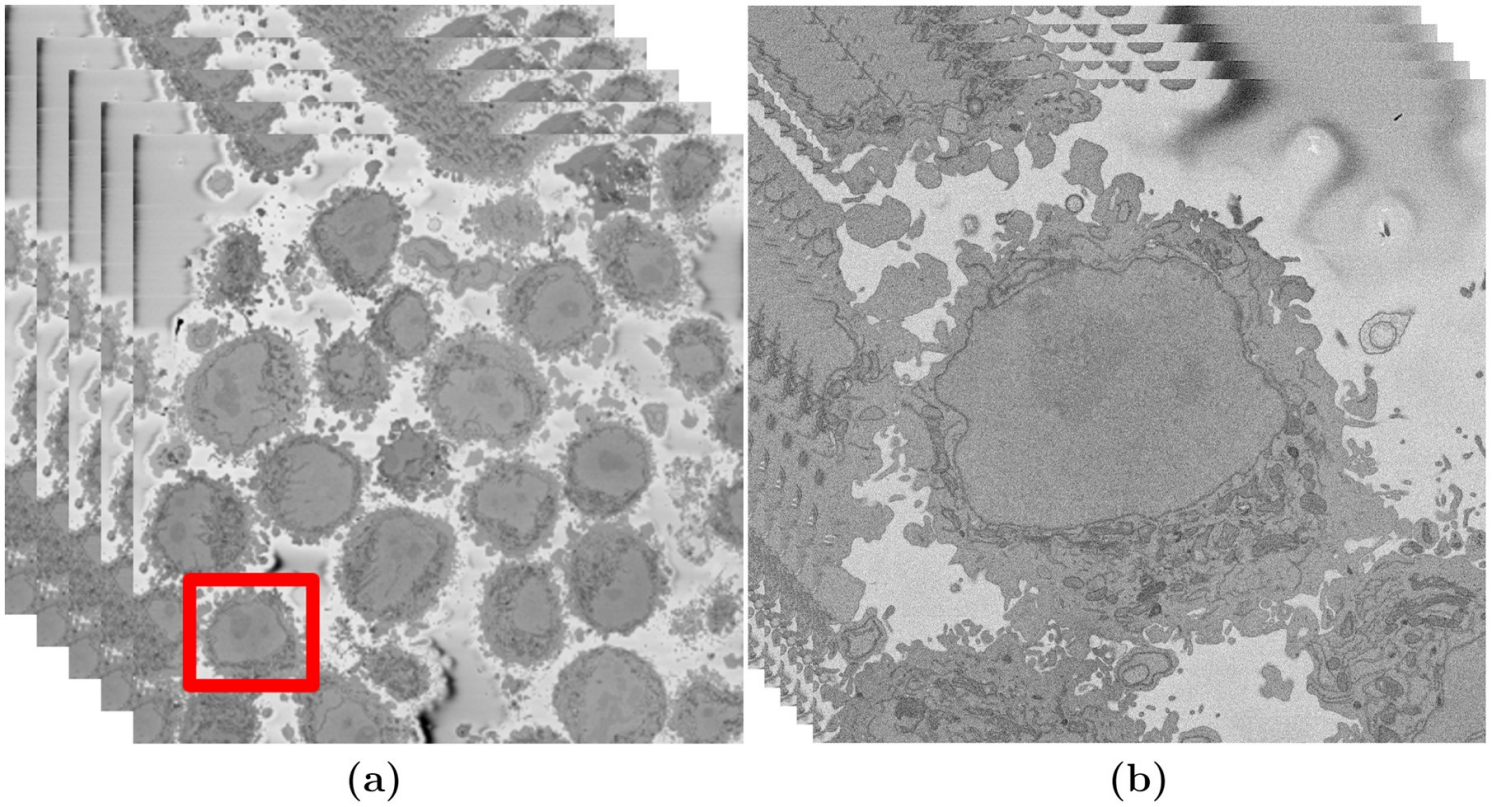
Illustration of the Serial Block Face Scanning Electron Microscope (SBF SEM) images containing HeLa cells. (a) Five representative 8192 × 8192 images arranged as 3D stack. The HeLa cells are the darker regions and the background is a brighter shade of grey. The red box indicates a region of interest (ROI)—a HeLa cell, that is magnified in (b). (b) Detail of the ROI with a single cell in the centre. This is slice 118 of 300. The nucleus is the large and fairly uniform region in the centre and it is surrounded by the nuclear envelope (NE) which is darker than the nucleus.

Once the cells were prepared, the samples were imaged using Serial Blockface Scanning Electron Microscopy (SBF SEM) with a 3View2XP (Gatan, Pleasanton, CA) attached to a Sigma VP SEM (Zeiss, Cambridge). The resolution of each image was 8, 192 × 8, 192 pixels corresponding to 10 × 10 nm (Fig 1a). In total, the sample was sliced 517 times and corresponding images were obtained. The slice separation was 50 nm. The images were acquired with high-bit contrast (16 bit) and after contrast/histogram adjustment, the intensity levels were reduced to 8 bit and therefore the intensity range was [0 − 255]. Then, one cell was manually cropped by selecting its estimated centroid and a volume of 2,000 × 2,000 × 300 voxels was selected (Fig 1b). Images are openly accessible via the EMPIAR public image database (http://dx.doi.org/10.6019/EMPIAR-10094).

### 2.3 Ground Truth (GT) and training data

**Fig 2:**
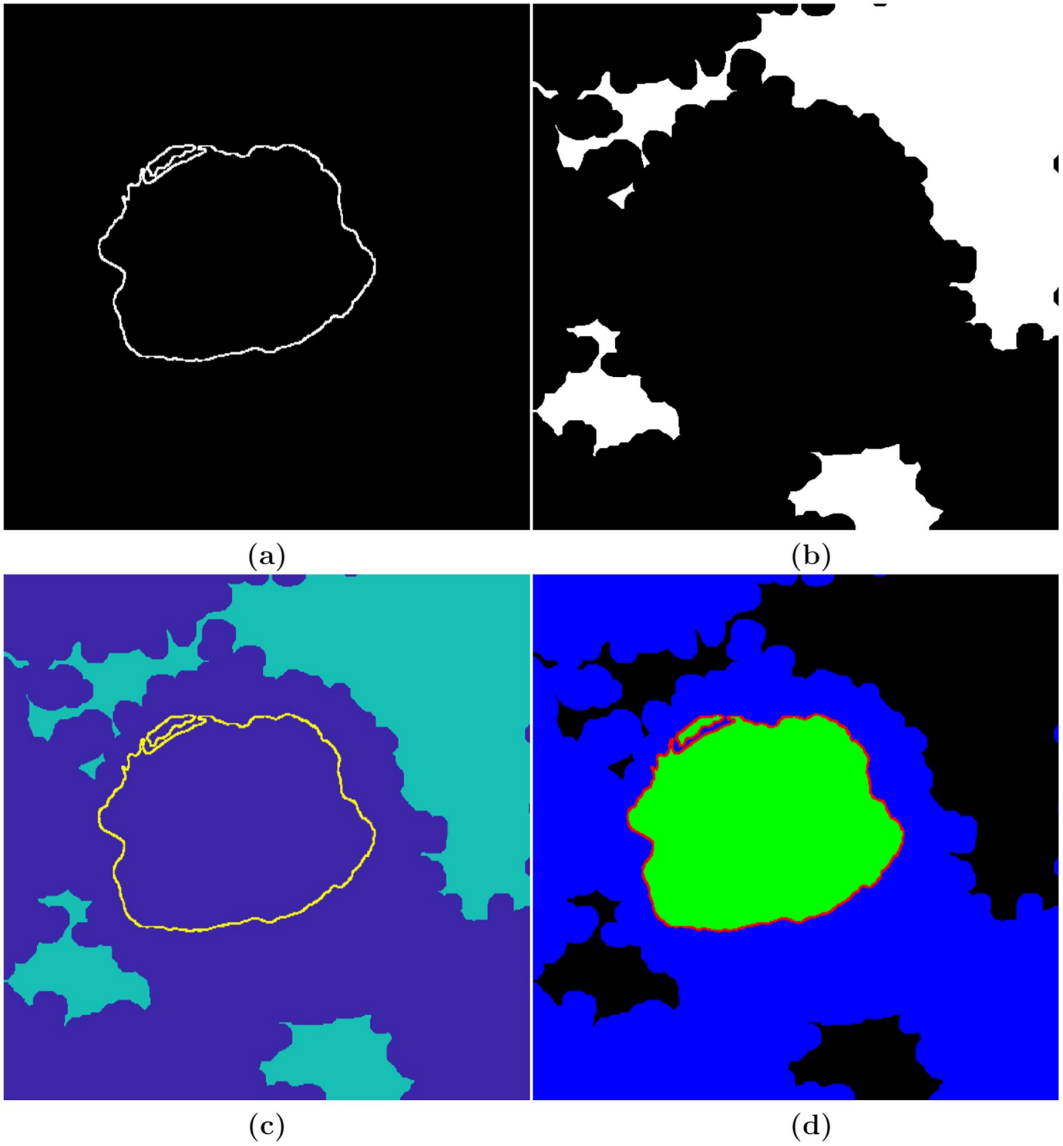
Illustration of the process followed to generate the ground truth. (a) Manually delineated nuclear envelope (NE). (b) Automatically segmented background. (c) Combination of the NE and the background. (d) Labelled image, generated in MATLAB^®^ *Image Labeler*, indicating the four different classes—nuclear envelope (red), nucleus (green), rest of the cell (blue), and background (black).

The three hundred slices were segmented with a combination of manual and algorithmic steps to provide a ground truth (GT). The NE was delineated manually using Amira (ThermoFisher Scientific, Waltham, MA, USA) and a Wacom (Kazo, Japan) Cintiq 24HD interactive pen display by one of the authors (A.E.W.) in approximately 30 hours (Fig 2a). In order to determine whether disjoint regions belong to the nucleus, the user scrolled up and down through neighbouring slices to check the connectivity of the regions. In a few cases, there were discontinuities in the line of the NE, and thus to morphological dilation was applied to ensure a closed contour.

The background of a HeLa cell image was segmented automatically with an image-processing algorithm, which assumed that the background was brighter than the cells. The HeLa images were low-pass filtered with a Gaussian kernel with size *h* = 7 and standard deviation *σ* = 2 to remove high frequency noise. Canny edge detection was used to detect abrupt changes of intensity—edges. In order to to connect disjoint edges, they were further dilated. The complement of the edges (i.e. the regions where the intensity was relatively uniform) was then labelled and its average intensity calculated. The background was selected as the brighter and larger regions previously segmented. Morphological operators were used to fill holes and close the regions for a more uniform background (Fig 2b).

Next, the NE and the background were combined (Fig 2c), exported to MATLAB^®^ *Image Labeler* with which four classes (nuclear envelope, nucleus, rest of the cell, background) were labelled (Fig 2d). The GT was replicated to create an image with three channels to be consistent with the RGB images commonly used with pre-trained neural networks.

Ideally, the number of elements should be balanced between classes. However, the sizes of the classes in the HeLa data set were imbalanced, which is a common issue in biomedical imaging, especially the NE was relatively small as compared with the other classes.

To improve training, class weighting was used to balance the classes. The pixel label counts computed earlier was used in order to calculate the median frequency class weights.

### 2.4 Semantic segmentation of HeLa cells

#### 2.4.1 Image-processing algorithm

**Fig 3:**
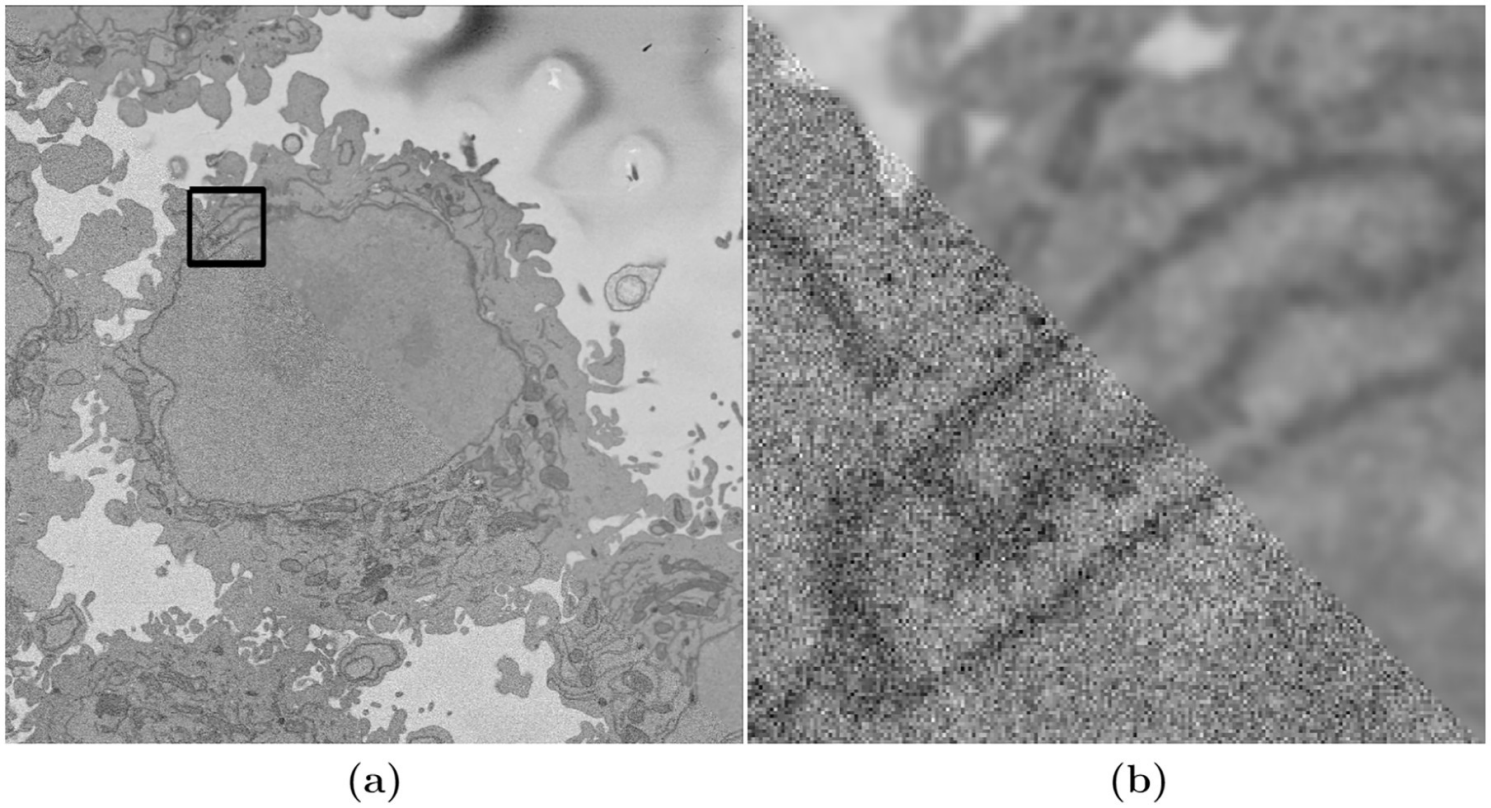
Illustration of the effects of low-pass filtering on one representative image of a HeLa cell. (a) Composite image where the lower-left triangle is original grainy image and the upper-right triangle has been low pass filtered. The box denotes a region of interest (ROI), which is magnified in (b). (b) Closer view of the composite image.

**Fig 4:**
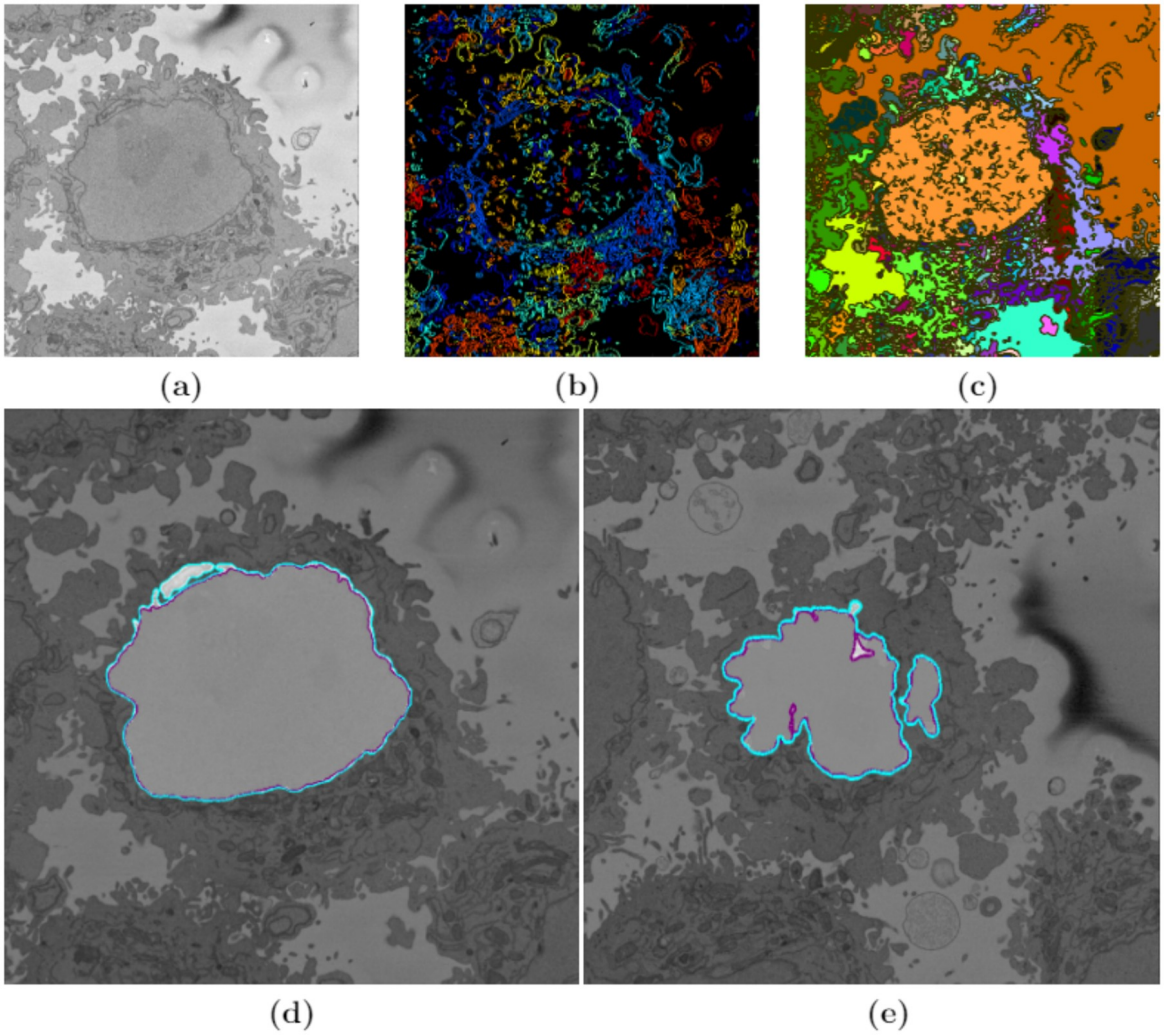
Illustration of intermediate steps of the proposed segmentation algorithm. (a) Cropped region around one HeLa cell (red box in Fig 1a and 1b), surrounded by resin (background) and edges of other cells. This image was low-pass filtered. (b) Edges detected by Canny algorithm. The edges were further dilated to connect those edges that may belong to the nuclear envelope (NE) but were disjoint due to the variations of the intensity of the envelope itself. (c) Superpixels obtained with the image-processing algorithm and they were generated by removing dilated edges. Small superpixels and those in contact with the image boundary were discarded and the remaining superpixels were smoothed and filled, before discarding those by size that did not belong to the nucleus. (d) Final segmentation of the NE overlaid on the filtered image shown in purple. The manual segmentation or the ground truth (GT) is also shown in cyan. (e) A different slice showing final segmentation and GT overlaid on the filtered image. By using neighbouring segmentation as input parameter to the current segmentation and taking the regions into account, the segmentation was considerably improved and was able to identify disjoint regions as part of a single nucleus. Details of differences can be appreciated and the nuclear area covered by the GT and segmentation was brightened up for visualisation purposes.

The initial step of the image-processing algorithm [75] filtered the images with a low-pass filter with a Gaussian kernel with size *h* = 7 and standard deviation *σ* = 2 to remove high frequencies and to enhance the larger scale edge features. This was required as the images presented a grainy texture, which would impact in subsequent steps (Fig 3a and 3b), which relied on the intensity of the classes. The algorithm then exploited the abrupt change in intensity at the NE compared with the neighbouring cytoplasm and nucleoplasm by applying Canny edge detection [79]. To connect any disjoint edges, these were dilated by calculating a distance map from the edges and then all pixels within a certain distance were included as edges. The minimum distance was 5 and could grow according to the standard deviation of the Canny edge detector, which is an input parameter of the algorithm. These disjoint edges were part of the NE and were initially disjoint due to intensity variations in the envelope itself (Fig 4b). The connected pixels not covered by the dilated edges were labelled by the standard 8-connected objects found in the image to create a series of superpixels (Fig 4c). The superpixel size was not restricted so that large superpixels covered the background and nucleoplasm. Morphological operators were used to: remove regions in contact with the borders of the image, remove small regions, fill holes inside larger regions and close the jagged edges. From the volumetric perspective, the algorithm began at the central slice of the cell, which was assumed to be the one in which the nuclear region would be centrally positioned and have the largest diameter. The algorithm exploited the 3D nature of the data by propagating the segmented NE of a slice to the adjacent slices (up and down). The NE of a previous slice was used to check the connectivity of disjoint regions or *islands* separate from the main nuclear region. This is the same strategy that a human operator would follow by considering contiguous images and proceeded in both directions (up and down through the neighbouring slices) and propagated the region labelled as the nucleus to decide if a disjoint nuclear region in the neighbouring slices (above or below) was connected above or below the current slice of analysis. When a segmented nuclear region overlapped with the previous nuclear segmentations, it was maintained, when there was no overlap, it was discarded (Fig 4d). A different slice with disjoint areas is shown after image processing algorithm segmentation in Fig 4e.

#### 2.4.2 Deep learning architectures for semantic segmentation—VGG16, ResNet18 and Inception-ResNet-v2 Net configurations

A typical CNN combines a series of layers: convolutional layers followed by sub-sampling layers (Pooling layer), then another convolutional layers followed by pooling layers, and can continue for a certain number of times after which fully-connected layers are added to produce a prediction (e.g. estimated class probabilities). This layer-wise arrangement allows CNNs to combine low-level features to form higher-level features, learn features and eliminate the need for hand crafted feature extractors. In addition, the learned features are translation invariant, incorporate the two-dimensional (2D) spatial structure of images which contributed to CNNs achieving state-of-the-art results in image-related tasks [80].

The input to a CNN, i.e. an image to be segmented classified, transits through the different layers to produce at the end some scores (one score per neuron in the last layer). These scores can be interpreted as the probability of the image to belong to each of the classes, which in this work are: nucleus, nuclear envelope, rest of the cell, and background. The goal of the training process is to learn the weights of the filters at the various layers of CNN. The output of one of the layers before the last layer, which is fully connected, can be used as a global descriptor for the input image. The descriptor can then be used for various image analysis tasks [48].

Three pre-trained deep learning architectures, VGG16, ResNet18 and Inception-ResNet-v2 were fine-tuned to perform semantic segmentation of HeLa cells imaged with SBF SEM. These pre-trained deep learning architectures have been widely explained in the literature, but for completeness, a brief description of each architecture is given below.

**Fig 5:**
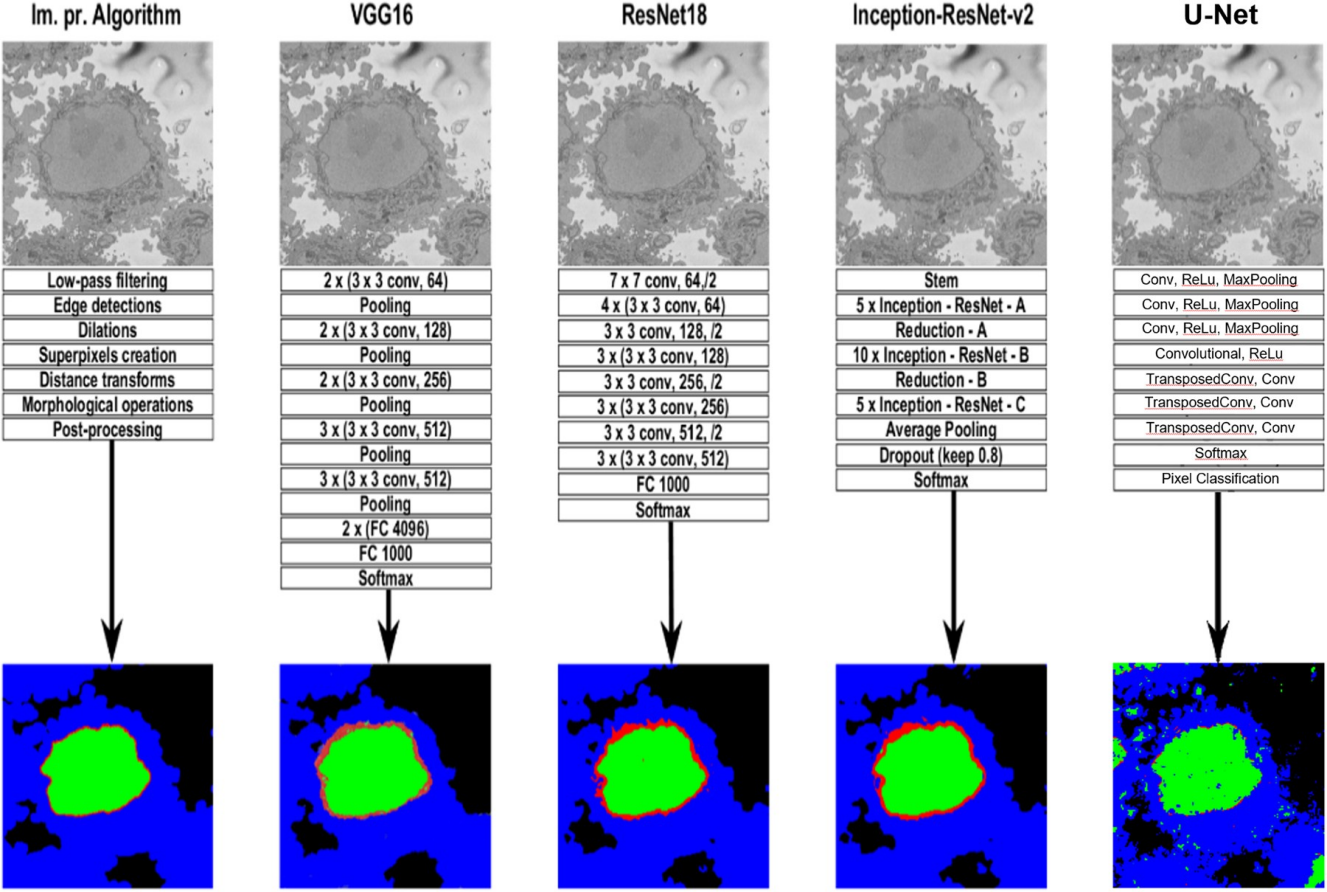
Graphical illustration of the five algorithms compared in this work. A representative slice of a HeLa cell image, the traditional image processing algorithm intermediate steps (first column), illustrations of four pre-trained deep learning architectures (Convolutional Neural Network (CNN))—VGG16 (second column), ResNet18 (third column), and Inception-ResNet-v2 (fourth column), and U-Net (last column) for the analysis of HeLa images, and semantic segmentation results showing four classes (nucleus, nuclear envelope, rest of the cell, and background) from all methodologies. A CNN is composed of an input layer, an output layer, and many hidden layers in between. These layers perform operations that alter the data with the intent of learning features specific to the data. Three of the most common layers are: convolution, activation or Rectified linear unit (ReLU), and pooling. An additional dropout layer is implemented in Inception-ResNet-v2 (4th column) in order to avoid over-fitting. In this work, a stack of convolutional layers is followed by Fully-Connected (FC) layers and the soft-max layer which performs a 4-way classification and thus contains 4 channels (one for each class—nucleus, nuclear envelope, rest of the cell, and background). These operations are repeated over tens or hundreds of layers with each layer learning to identify different features.

*VGG16*. VGG16 is a convolution neural network (CNN) [46, 48], which takes as input 224 × 224 RGB images. The image is passed through a stack of convolutional layers, where filters are used with a very small receptive field: 3 × 3 (which is the smallest size to capture the notion of left/right, up/down, centre). In one of the configurations 1 × 1 convolution filters are utilised, which can be seen as a linear transformation of the input channels (followed by non-linearity). The convolution stride is fixed to 1 pixel; the spatial padding of convolution layer input is such that the spatial resolution is preserved after convolution, i.e. the padding is 1 pixel for 3 × 3 convolution layers. Spatial pooling is carried out by five max-pooling layers, which follow some of the convolution layers (not all the convolution layers are followed by max-pooling). Max-pooling is performed over a 2 × 2 pixel window, with stride 2. A stack of convolutional layers (which has a different depth in different architectures) is followed by three Fully-Connected (FC) layers: the first two have 4096 channels each, the third performs 1000-way ILSVRC classification and thus contains 1000 channels (one for each class). The final layer is the soft-max layer. The configuration of the fully connected layers is the same in all networks. All hidden layers are equipped with the rectification (ReLU [44]) non-linearity. The second column of Fig 5 shows the basic network architecture of VGG16.

*ResNet18*. ResNet18 is mainly inspired by the philosophy of VGG16 [70], its total number of weighted layers is 18 and has an image input size of 224-by-224. The convolutional layers mostly have 3 × 3 filters and follow two simple design rules: (i) for the same output feature map size, the layers have the same number of filters; and (ii) if the feature map size is halved, the number of filters is doubled so as to preserve the time complexity per layer. Shortcut connections which turn the network into its counterpart residual version are inserted. The identity shortcuts can be directly used when the input and output are of the same dimensions. When the dimensions increase, two options are considered: (a) The shortcut still performs identity mapping, with extra zero entries padded for increasing dimensions. This option introduces no extra parameter; (b) The projection shortcut is used to match dimensions (done by 1 × 1 convolutions). For both options, when the shortcuts go across feature maps of two sizes, they are performed with a stride of 2. Down sampling is performed directly by convolutional layers that have a stride of 2. The network ends with a global average pooling layer and a 1000-way fully-connected layer with soft-max. ResNet18 has fewer filters and lower complexity than VGG16. The third column of Fig 5 shows the basic network architecture of ResNet18.

*Inception-ResNet-v2*. The Inception deep convolutional architecture was introduced as GoogLeNet in [81] and named Inception-v1. Later the Inception architecture was refined in various ways, first by the introduction of batch normalisation [82] (Inception-v2). Later by additional factorisation ideas in the third iteration [83] which is referred to as Inception-v3. The Inception architecture is highly tunable, meaning that there are a lot of possible changes to the number of filters in the various layers that do not affect the quality of the fully trained network. The introduction of residual connections leads to dramatically improved training speed for the Inception architecture. For the residual versions of the Inception networks, Inception blocks in which a 5 × 5 convolution is replaced two 3 × 3 convolution operations to improve computational speed are used—stacking two 3 × 3 convolutions leads to a boost in performance. Each Inception block is followed by filter-expansion layer (1 × 1 convolution without activation) which is used for scaling up the dimensionality of the filter bank before the residual addition to match the depth of the input. This is needed to compensate for the dimensionality reduction induced by the Inception block.

Several versions of the residual version of Inception were tried. The first version, Inception-ResNet-v1, has roughly the computational cost of Inception-v3, while Inception-ResNet-v2 matches the raw cost of the newly introduced Inception-v4 network. However, the step time of Inception-v4 proved to be significantly slower in practice, probably due to the larger number of layers. The models Inception-v3 and Inception-v4 are deep convolutional networks not utilising residual connections while Inception-ResNet-v1 and Inception-ResNet-v2 are Inception style networks that utilise residual connections instead of filter concatenation.

Inception-ResNet-v2 is the combination of two of residual connections and the latest revised version of the Inception architecture [71] and it has an image input size of 299-by-299 and 164 layers deep. In the Inception-ResNet block, multiple sized convolutional filters are combined by residual connections. The usage of residual connections not only avoids the degradation problem caused by deep structures but also reduces the training time [84]. The 35 × 35, 17 × 17 and 8 × 8 grid modules, known as Inception-A, Inception-B and Inception-C blocks, are used in the Inception-ResNet-v2 network. The last column of Fig 5 shows the basic network architecture of Inception-ResNet-v2.

Both ResNet18 and Inception-ResNet-v2 were fined tuned and trained on a new segmentation task—HeLa cells with four different classes. ResNet18 and Inception-ResNet-v2 are Directed Acyclic Graph (DAG) networks with branches that are faster, smaller, and more accurate. ResNet18 has about 11.7 million (approx) parameters while Inception-ResNet-v2 has about 55.9 million (approx) parameters. ResNet18 and Inception-ResNet-v2 are convolutional neural networks that were trained on more than a million images from the ImageNet [85] database. They can classify images into 1000 object categories, such as keyboard, mouse, pencil, and many animals. As a result, the networks have learned rich feature representations for a wide range of images.

*U-Net*. U-Net is convolutional network architecture with broad application and has become a broadly used tool for semantic segmentation. U-Net architecture is a type of fully convolutional network [86] in which, after the downsampling steps obtained by convolutions and downsampling, there is a series of upsampling steps through which the classification is propagated towards higher resolution layers and finally returns to the original resolution of the input. The shape of the architecture is more or less symmetric with the shape of the letter “U”, hence the name. The U-Net can be trained end-to-end from relatively few pairs or patches of images and their corresponding classes. Applications of U-Nets include cell counting, detection, and morphometry, [87], automatic brain tumour detection and segmentation [88] and texture segmentation [89].

The U-Net architecture was constructed with the following layers: Input, Convolutional, ReLu, Max Pooling, Transposed Convolutional, Convolutional, Softmax and Pixel Classification. Several training configurations were tested, number of layers (10,15,20), number of epochs (5,10,15), classifiers stochastic gradient descent (sgdm), Adam (Adam) [90] and Root Mean Square Propagation (RMSprop) and size of training images (64 × 64, 128 × 128) and the best results were obtained with 36,000 training pairs, 15 epochs, 20 layers and RMSprop with 128 × 128 training images. The downsampling and upsampling blocks had the following configuration:

- Input,
- Convolutional, ReLu, Max Pooling,
- Convolutional, ReLu, Max Pooling,
- Convolutional, ReLu, Max Pooling,
- Convolutional, ReLu,
- Transposed Convolutional, Convolutional,
- Transposed Convolutional, Convolutional,
- Transposed Convolutional, Convolutional,
- Softmax,
- Pixel Classification.

### 2.5 Description of network training

The HeLa Pixel-Labeled Images data set, shown in Fig 2d, provides pixel-level labels for four semantic classes including the nucleus, nuclear envelope, rest of the cell, and background. These classes were specified before the training. The images and labelled training data in the HeLa data set are 2000 × 2000 × 1. In order to reduce training time and memory usage, all images and pixel label images were resized to 360 × 480 × 3. The network was trained using 60% of the images from the data set. The rest of the images (40%) were used to test the network after training. The network randomly splits the image and pixel label data into a training and test set. The whole data set for each pattern has been divided into two. Sixty percent is kept for training and its number has been increased artificially by using image augmentation techniques such as translation and reflections. EM images do not contain any colour information therefore they have the dimensions (*n*_*h*_, *n*_*w*_, *n*_*d*_) = (2000, 2000, 1). As all three deep learning architectures (VGG16, ResNet18 and, Inception-ResNet-v2) expect an image with *n*_*d*_ = 3, the other 2 dimensions were a copy of the first dimension creating a greyscale image before training the algorithm.

The training took 21.25 hours on a single CPU and the training plot was obtained to check the accuracy and loss during training of the three pre-trained deep learning architectures (VGG16, ResNet18, and Inception-ResNet-v2). The optimisation algorithm used for training is stochastic gradient descent with momentum (sgdm) and this was specified in training options. The sgdm algorithm can oscillate along the path of steepest descent towards the optimum. Adding a momentum term to the parameter update is one way to reduce this oscillation [91].

An image data augmenter in neural network configures a set of pre-processing options for image augmentation, such as resizing, rotation, and reflection and generates batches of augmented images. Data augmentation is used during training to provide more examples to the network because it helps improve the accuracy of the network, prevents the network from over fitting [54] and memorising the exact details of the training images. Three hundred images were used for training. These were split 60%/40% for training and testing. Data augmentation was applied with random translations of ±10 pixels on the horizontal and vertical axes and random reflection on the horizontal axis.

The training data and data augmentation selections were combined. The deep network reads batches of training data, applies data augmentation, and sends the augmented data to the training algorithm.

The VGG16 deep network training had 100 epochs with learning rate 0.001. Iterations per epoch were 45 therefore the total number of iterations for the whole training was 4500. Similarly, training took approximately 1.5 hours for ResNet18 and 4.7 hours for Inception-ResNet-v2 with 30 epochs and 660 iterations and learning rate 0.00009 on a single CPU.

The semantic segmentation results from image-processing algorithm, VGG16, ResNet18, and Inception-ResNet-v2 are shown in Fig 5. These results were compared with the labelled data shown in (Fig 2d) and accuracy and Jaccard similarity index were calculated to assess the accuracy of the network. In order to measure accuracy for the data set, deep learning architectures were run on the entire test set.

**Fig 6:**
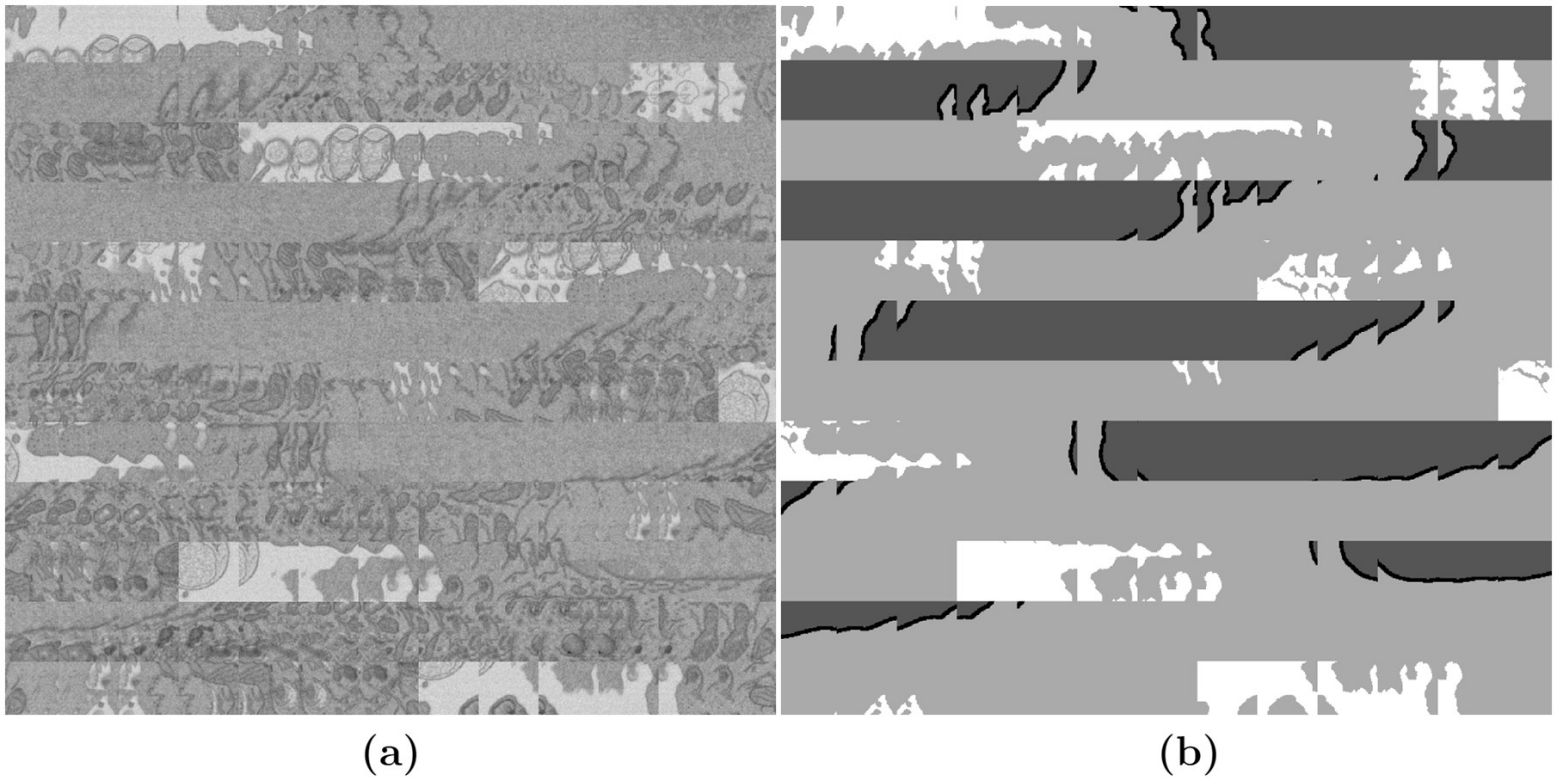
Illustration of the image-label pairs created to train the U-Net architecture. (**a**) A sample of regions, each 128 × 128 pixels placed next to each other as a montage. (**b**) Montage of the labels corresponding to the regions of (**a**). The labels contain four classes, from dark to bright: Nuclear Envelope, Nucleus, Cell, Background.

To train the U-Net, the image input layer was configured for the 128 × 128 patches. The patches were formed from a subset of the images, specifically the odd slices between slice 101 and slice 180. The images were cropped to regions of 128×128 pixels with an overlap of 50%. An illustration of the data and the labels is shown in Fig 6.

### 2.6 Quantitative comparison

**Fig 7:**
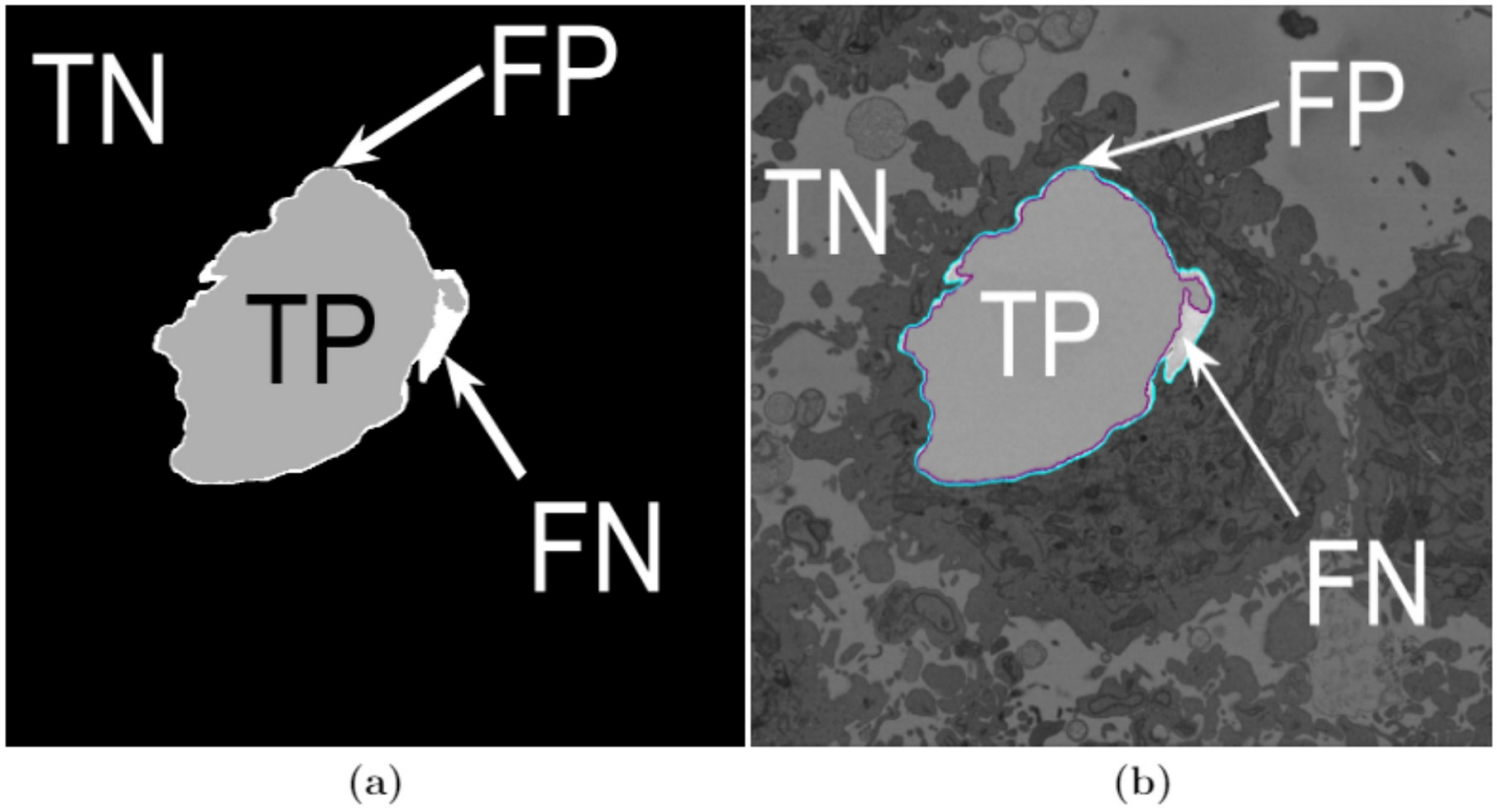
Illustration of the pixel-based metrics. (a) True Positives (TP, nuclear pixels segmented as nucleus), true negatives (TN, background pixels segmented as background), false positives (FP, background pixels segmented as nucleus) and false negatives (FN, nuclear pixels segmented as background). These quantities were used to compute accuracy and Jaccard similarity index for the image-processing algorithm and three pre-trained deep learning architectures. (b) Segmentation overlaid on the same slice as (a) (slice 184/300—on a filtered image): ground truth (GT) in cyan, automated segmentation in purple.

In order to evaluate the accuracy of the image processing segmentation algorithm and deep learning architectures, two different pixel-based metrics were used: accuracy and Jaccard similarity index, or simply Jaccard index (JI) [92], were calculated. Both metrics arise from the allocation of classes to every pixel of an image, for which four cases exist: (i) true positive (TP), correspond to pixels which were correctly predicted as a certain class (e.g. nucleus) or to have a condition present (e.g. a disease), (ii) true negative, (TN) corresponds to a pixel that was correctly predicted to be background or for which the condition not present (i.e. negative), (iii) false positive, (FP) correspond to those pixels predicted to be a class (or to have a condition) but correspond to background (or not have the condition), and (iv) false negative (FN), correspond to those pixels that were predicted to be background (or not have the condition) but in reality belong to a class (or have the condition). Fig 7 illustrates these cases for a sample slice of the data. Thus, accuracy can be defined mathematically in the following way:

$$\begin{array}{c} \text{Accuracy}=\frac{TP+TN}{TP+FN+TN+FP} \end{array}$$ (1)

which corresponds to the sum of all correctly predicted pixels over the total number of pixels. Similarly, Jaccard index is calculated as:

$$\begin{array}{c} \text{Jaccard}\;\text{Similarity}\;\text{Index}=\frac{TP}{TP+FP+FN} \end{array}$$ (2)

In both cases, the higher the number of the metric, the better the segmentation. It should be noticed that Jaccard is more rigorous as it does not take into account TN or background pixels, which in those cases where objects of interest are small in comparison with the image can bias poor results to have high accuracy. The metrics were calculated on a per-slice basis for all algorithms.

## 3 Results

**Fig 8:**
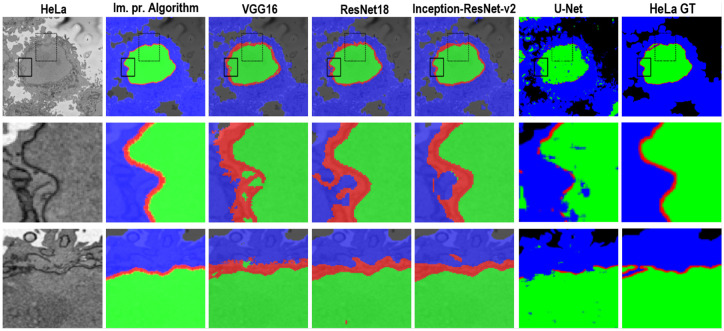
A representative HeLa image, semantic segmentation results overlaid on the filtered image and the ground truth. Top row: Boxes denote two Region of Interests (ROIs) on filtered image as well as semantic segmentation results. Results were obtained from all algorithms—a traditional and four deep learning architectures. The ROIs are magnified and shown in the middle and bottom rows. Middle row: ROI 1 corresponding black box with solid line. Bottom row: ROI 2—box with dashed line. Differences in segmentation results can be seen from these ROIs—i.e. the red dot obtained from ResNet18 semantic segmentation on bottom row and 4^*th*^ column.

In this work, images of HeLa cells observed with SBF SEM were semantically segmented with an image-processing algorithm and three pre-trained deep learning architectures (convolutional neural networks—CNNs), VGG16, ResNet18, and Inception-ResNet-v2. The accuracy and Jaccard similarity index of the segmentations against a ground truth were calculated. The image-processing algorithm is fully automatic and processed each slice of the HeLa cells in approximately 8 seconds and a whole cell containing 300 slices in approximately 40 minutes. On the other hand, the deep neural network architectures were fine-tuned and trained in 21.25 hours (VGG16), 1.5 hours (ResNet18), and 4.7 hours (Inception-ResNet-v2) to perform semantic segmentation on the whole cell with relatively good accuracy and differences can be appreciated in Fig 8 with two ROIs.

**Fig 9:**
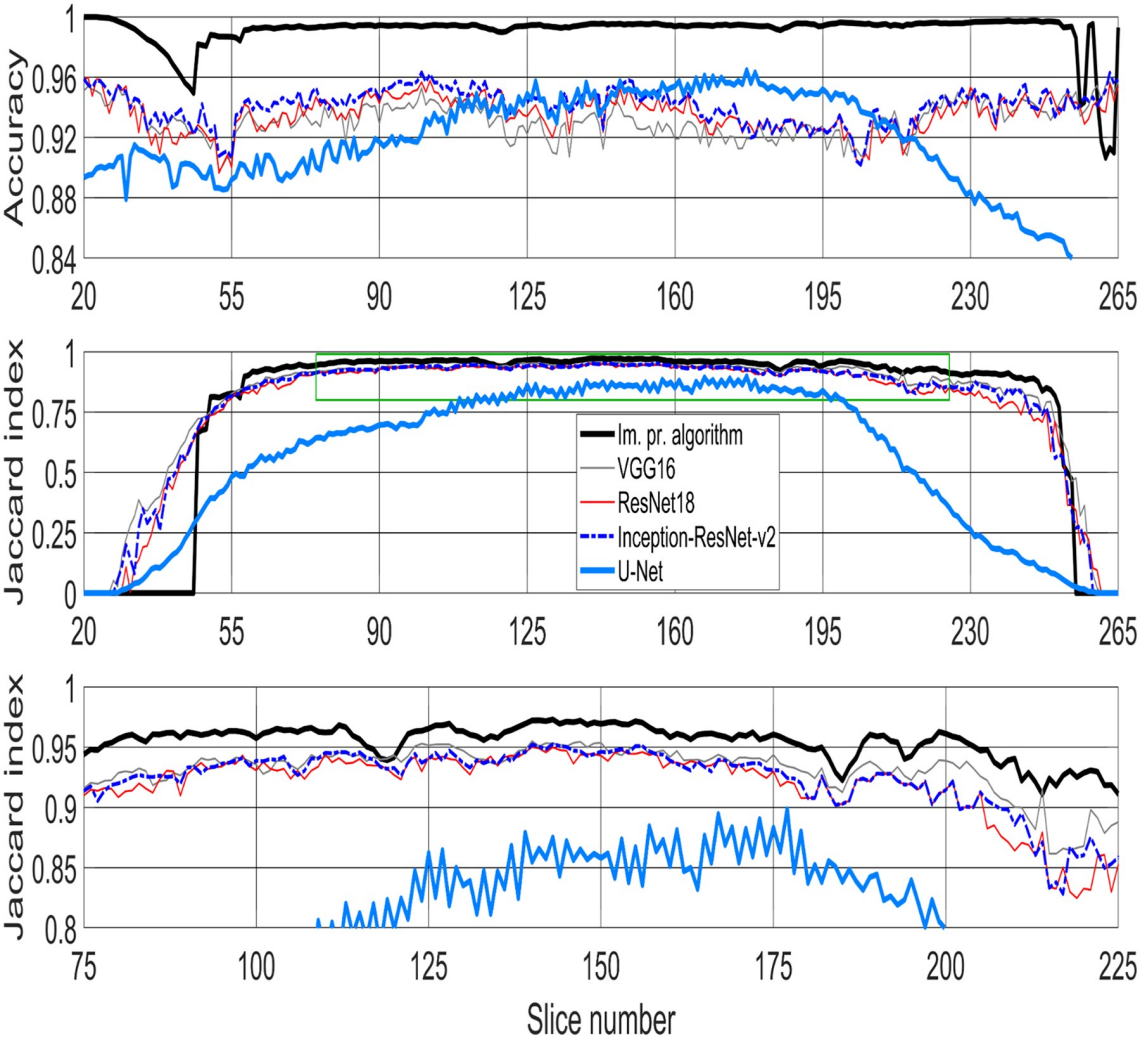
Metrics used for comparison between image-processing algorithm and four pre-trained deep learning architectures—VGG16, ResNet18, Inception-ResNet-v2, and U-Net for semantic segmentation of HeLa cell imaged with Electron Microscopy (EM). (Top row) Accuracy. (Middle row) Jaccard similarity index, also known as intersection over union, for all algorithms. Green box denotes the central slices and corresponding Jaccard similarity index that is magnified below. (Bottom row) Jaccard similarity index for central slices (slices between 75/300 and 225/300—interquartile range (IQR)), for easier comparison. The image-processing algorithm outperforms all deep learning architectures.

Segmentation of the nuclear envelope by all four deep learning architectures was outperformed by the image-processing algorithm as shown in Figs 8 and 9. Visually, the semantic segmentation results overlap well for classes such as the nucleus, the rest of the cell, and background. However, smaller objects like the nuclear envelope are not as accurate. Although the overall data set performance is quite high, the class metrics show that under represented classes such as nuclear envelope is not segmented as well as classes such as the nucleus, the rest of the cell, and background.

## 4 Discussion

In this paper, a classical and unsupervised image processing algorithm was used to perform semantic segmentation of cancerous HeLa cell images from SBF SEM and compared with four deep neural network architectures. The first pre-trained deep neural network architectures, VGG16, ResNet18 and Inception-ResNet-v2 were trained in ImageNet and fine-tuned for semantic segmentation of the HeLa cells. The U-Net architecture was trained from scratch constructing image-label pairs. Four different classes, nucleus, nuclear envelope, the rest of the cell, and background were used in labelling training data for deep learning architectures. Two similarity metrics, accuracy and Jaccard index, were calculated so the image-processing algorithm was compared with deep learning architectures. For the central slices, i.e. the slices between 75/300 and 225/300, the image-processing algorithm outperformed all deep learning architectures in both accuracy and Jaccard similarity Index (Fig 9). The results on the bottom and top slices were mixed, higher accuracy for image-processing and higher Jaccard for deep-learning. As the nucleus was not present in the bottom 26 and the top 40 slices, there was a larger background and thus the accuracy remained high for all techniques, i.e. the TN kept the metric high. On the other hand, the Jaccard index decreased towards the extremes for all techniques due to the smaller regions of the nucleus. The decrease was most sharp for the image processing algorithm as it failed to detect nuclei below and above a certain level and this is most likely due to some parameters of the algorithm.

**Fig 10:**
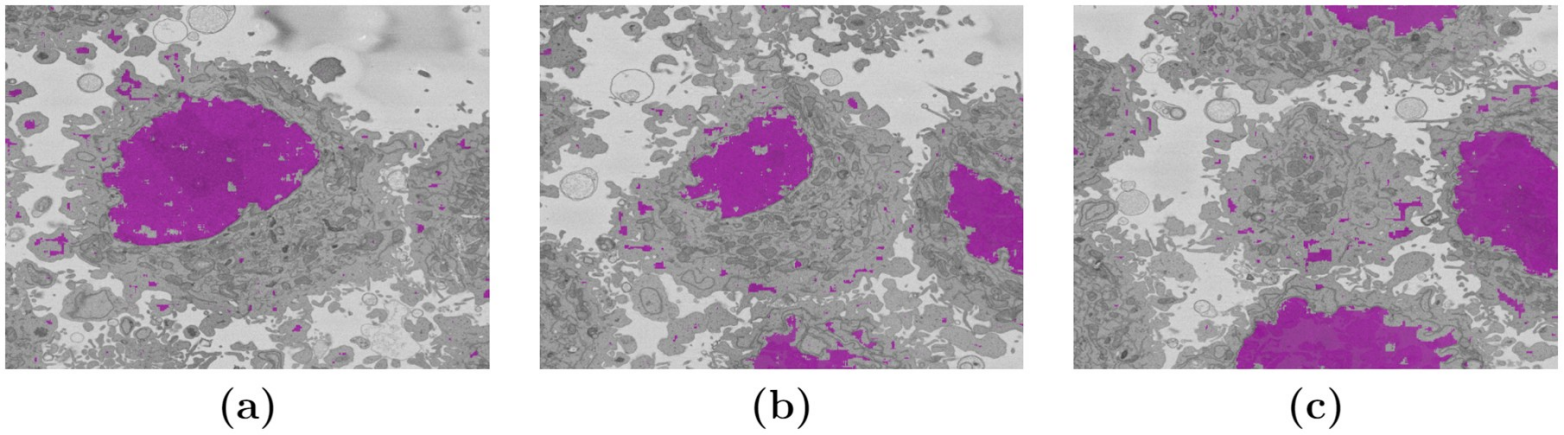
Three slices semantically segmented slices with U-Net. (a) Slice 170, towards the centre of the cell, accuracy = 0.954, Jaccard Index = 0.854. (b) Slice 220, towards the top of the cell, accuracy = 0.906, Jaccard Index = 0.435. (c) Slice 260, on the very top of the cell, accuracy = 0.818, Jaccard Index = 0.0007. The area of the nucleus has been highlighted in pink. It should be noticed that in (a) the nuclear area is well segmented but there are other artefactual segmentations. In (b,c) the nuclei of contiguous cells are also segmented. These detections will drive down both accuracy and Jaccard Index.

The results provided by the U-Net semantic segmentation were very interesting. The training of the U-Net provided sufficient samples for the network to distinguish the nuclei of cells, that is, not only of the cell that is located in the centre but also of other nuclei visible within the slices (Fig 10). It should be remembered that one assumption of the segmentation task was that there was a single cell in the centre of the volume of interest. This assumption will impact on the results of the U-Net as the ground truth was constructed with a single nucleus in the region. Therefore, for slices closer to the centre the accuracy and Jaccard Index were higher, and as the central nucleus became smaller towards the edges, and other nuclei appeared, these metrics decreased. The construction of a separate ground truth for U-Net, which would reveal a more accurate comparison for U-Net, is beyond the scope of this paper.

Additional data that includes more samples of the under represented classes, nuclear envelope, in this case, might help improve the results as the sizes of the classes in the HeLa data set were imbalanced. For the image-processing algorithm, this would be expected, as the NE is more irregular on the top and bottom slices than on the central ones.

For the image-processing algorithm the segmentation of each cropped cell is fully automatic, unsupervised and the algorithm segments one slice in approximately 8 seconds and one whole cell in approximately 40 minutes.

As JI does not count true negatives (TN), the values decrease towards the top and bottom slices of the cells as the structure was considerably more complex and the areas become much smaller (Fig 9 (Middle row) and (Bottom row)). On the other hand, accuracy includes the TN in both numerator and denominator and this, especially in cases where the objects of interest are small and there are large areas of background (e.g. the top and bottom slices of the cell) would render very high accuracy. Therefore, in contrast to JI, accuracy increases in slices towards both top and bottom ends. Overall, the best results were obtained by the image processing segmentation algorithm especially for central slices—slices between 75/300 and 225/300 (Fig 9 (Bottom row)).

Deploying deep learning architectures [37] and training to learn patterns and features directly from EM images and to automatically segment the NE and other parts of a cell is indeed necessary as this will reduce the effort, assessment variability and provide a second opinion to support biomedical researchers’ decisions as it shortens the time required to segment the cell.

The main limitations of the algorithms are as follows. For the traditional image processing algorithm, it was assumed that there was a single HeLa cell of interest, which may be surrounded by fragments of other cells, but the centre of the cell of interest is located at the centre of a three-dimensional (3D) stack of images. In addition, it was assumed that the nuclear envelope was darker than the nuclei or its surroundings and that the background was brighter than any cellular structure. The limitations of the deep learning strategies were the training data. In the case of U-Net, 36000 pairs of images and labels were used, perhaps with a larger number the results would improve. Similarly, different configuration of the network, like number of epochs, could impact on the results.

The results of the deep learning approaches, for all cases, could have been improved by applying post-processing, e.g. to remove small regions of one class that were inside a large region of a different class, or by thinning or dilating the nuclear envelope. However, as the objective was to compare the image processing algorithm with the deep learning architectures, it was preferred not to post-process the latter ones. In addition, for the case of U-Net, the ground truth was restricted to a single nucleus in the volume. A ground truth with several nuclei could provide better results.

The main contributions of this work are: (a) Five segmentation strategies, one traditional image processing and four deep learning, have been objectively compared through the semantic segmentation of three hundred images of a HeLa cell. (b) The code developed for the segmentation strategies has been made available through GitHub. All the programming was performed in Matlab^®^ (The Mathworks^™^, Natick, USA). (c) The four-class ground truth for 300 slices has been created and made available through Zenodo.

## Supporting information

S1 Code (TXT)

## References

[1] Ribatti D. An historical note on the cell theory. Experimental Cell Research. 2018;364(1):1–4. 10.1016/j.yexcr.2018.01.038 29391153

[2] Candia J, Maunu R, Driscoll M, Biancotto A, Dagur P, McCoy JP, et al From Cellular Characteristics to Disease Diagnosis: Uncovering Phenotypes with Supercells. PLoS Computational Biology. 2013;9(9):e1003215 10.1371/journal.pcbi.1003215 24039568 PMC3763994

[3] Candia J, Banavar JR, Losert W. Understanding health and disease with multidimensional single-cell methods. Journal of Physics Condensed Matter: An Institute of Physics Journal. 2014;26(7):073102 10.1088/0953-8984/26/7/073102 24451406 PMC4020281

[4] Zhao J. Cell individuality: a basic multicellular phenomenon and its role in the pathogenesis of disease. Medical Hypotheses. 1995;44(5):400–402. 10.1016/0306-9877(95)90267-8 8583971

[5] Zhao J. A liability theory of disease: the foundation of cell population pathology. Medical Hypotheses. 1997;48(4):341–346. 10.1016/S0306-9877(97)90104-3 9160289

[6] Orrenius S. Apoptosis: molecular mechanisms and implications for human disease. Journal of Internal Medicine. 1995;237(6):529–536. 10.1111/j.1365-2796.1995.tb00881.x 7782723

[7] Prame Kumar K, Nicholls AJ, Wong CHY. Partners in crime: neutrophils and monocytes/macrophages in inflammation and disease. Cell and Tissue Research. 2018;371(3):551–565. 10.1007/s00441-017-2753-2 29387942 PMC5820413

[8] Peddie CJ, Collinson LM. Exploring the third dimension: Volume electron microscopy comes of age. Micron. 2014;61:9–19. 10.1016/j.micron.2014.01.009 24792442

[9] Denk W, Horstmann H. Serial block-face scanning electron microscopy to reconstruct three-dimensional tissue nanostructure. PLoS Biol. 2004;2(11). 10.1371/journal.pbio.0020329 15514700 PMC524270

[10] De Magistris P, Antonin W. The Dynamic Nature of the Nuclear Envelope. Current biology: CB. 2018;28(8):R487–R497. 10.1016/j.cub.2018.01.073 29689232

[11] Wilson KL, Berk JM. The nuclear envelope at a glance. J Cell Sci. 2010;123(12):1973–1978. 10.1242/jcs.019042 20519579 PMC2880010

[12] De Magistris P, Antonin W. The dynamic nature of the nuclear envelope. Current Biology. 2018;28(8):R487–R497. 10.1016/j.cub.2018.01.073 29689232

[13] Hetzer MW. The nuclear envelope. Cold Spring Harbor perspectives in biology. 2010;2(3):a000539 10.1101/cshperspect.a000539 20300205 PMC2829960

[14] Georgatos SD. The inner nuclear membrane: simple, or very complex? The EMBO journal. 2001;20(12):2989–2994. 10.1093/emboj/20.12.2989 11406575 PMC150211

[15] Hennig T, O’Hare P. Viruses and the nuclear envelope. Current opinion in cell biology. 2015;34:113–121. 10.1016/j.ceb.2015.06.002 26121672

[16] Bhargava A, Lahaye X, Manel N. Let me in: Control of HIV nuclear entry at the nuclear envelope. Cytokine & growth factor reviews. 2018;40:59–67. 10.1016/j.cytogfr.2018.02.006 29526438

[17] Snoussi K, Kann M. Interaction of parvoviruses with the nuclear envelope. Advances in biological regulation. 2014;54:39–49. 10.1016/j.jbior.2013.09.008 24157125

[18] Mettenleiter TC. Breaching the barrier—The nuclear envelope in virus infection. Journal of molecular biology. 2016;428(10):1949–1961. 10.1016/j.jmb.2015.10.001 26522933

[19] Cohen S, Etingov I, Panté N. Effect of viral infection on the nuclear envelope and nuclear pore complex. In: International review of cell and molecular biology. vol. 299 Elsevier; 2012 p. 117–159. 10.1016/B978-0-12-394310-1.00003-5 22959302

[20] Roux KJ, Burke B. Nuclear envelope defects in muscular dystrophy. Biochimica et Biophysica Acta (BBA)-Molecular Basis of Disease. 2007;1772(2):118–127. 10.1016/j.bbadis.2006.06.001 16904876

[21] Chow KH, Factor RE, Ullman KS. The nuclear envelope environment and its cancer connections. Nature Reviews Cancer. 2012;12(3):196 10.1038/nrc3219 22337151 PMC4338998

[22] Malhas AN, Vaux DJ. Nuclear envelope invaginations and cancer. In: Cancer Biology and the Nuclear Envelope. Springer; 2014 p. 523–535. 10.1007/978-1-4899-8032-8_24 24563364

[23] Lim S, Quinton RJ, Ganem NJ. Nuclear envelope rupture drives genome instability in cancer. Molecular biology of the cell. 2016;27(21):3210–3213. 10.1091/mbc.E16-02-0098 27799497 PMC5170854

[24] Jose I, Batrakou DG, Schirmer EC. Cancer biology and the nuclear envelope: a convoluted relationship. In: Seminars in cancer biology. vol. 23 (2). Elsevier; 2013 p. 125–137. 10.1016/j.semcancer.2012.01.008 22311402

[25] Alvarado-Kristensson M, Rosselló CA. The Biology of the Nuclear Envelope and Its Implications in Cancer Biology. International journal of molecular sciences. 2019;20(10):2586 10.3390/ijms20102586 31137762 PMC6566445

[26] Vidal C, Bermeo S, Fatkin D, Duque G. Role of the nuclear envelope in the pathogenesis of age-related bone loss and osteoporosis. BoneKEy reports. 2012;1 10.1038/bonekey.2012.62 23951459 PMC3727739

[27] Burke B, Mounkes LC, Stewart CL. The nuclear envelope in muscular dystrophy and cardiovascular diseases. Traffic. 2001;2(10):675–683. 10.1034/j.1600-0854.2001.21001.x 11576443

[28] Zhou C, Li C, Zhou B, Sun H, Koullourou V, Holt I, et al Novel nesprin-1 mutations associated with dilated cardiomyopathy cause nuclear envelope disruption and defects in myogenesis. Human molecular genetics. 2017;26(12):2258–2276. 10.1093/hmg/ddx116 28398466 PMC5458344

[29] Weintraub RG, Semsarian C, Macdonald P. Dilated cardiomyopathy. The Lancet. 2017;390(10092):400–414. 10.1016/S0140-6736(16)31713-5 28190577

[30] Dobrzynska A, Gonzalo S, Shanahan C, Askjaer P. The nuclear lamina in health and disease. Nucleus. 2016;7(3):233–248. 10.1080/19491034.2016.1183848 27158763 PMC4991244

[31] Bonne G. Nuclear envelope proteins in health and diseases. In: Seminars in cell & developmental biology. vol. 29; 2014 p. 93 10.1016/j.semcdb.2014.04.023 24755165

[32] Worman HJ, Östlund C, Wang Y. Diseases of the nuclear envelope. Cold Spring Harbor perspectives in biology. 2010;2(2):a000760 10.1101/cshperspect.a000760 20182615 PMC2828284

[33] Robijns J, Houthaeve G, Braeckmans K, De Vos WH. Loss of nuclear envelope integrity in aging and disease. In: International review of cell and molecular biology. vol. 336 Elsevier; 2018 p. 205–222. 10.1016/bs.ircmb.2017.07.013 29413891

[34] Cau P, Navarro C, Harhouri K, Roll P, Sigaudy S, Kaspi E, et al Nuclear matrix, nuclear envelope and premature aging syndromes in a translational research perspective. In: Seminars in cell & developmental biology. vol. 29 Elsevier; 2014 p. 125–147. 10.1016/j.semcdb.2014.03.021 24662892

[35] Ahmed MS, Ikram S, Bibi N, Mir A. Hutchinson–gilford progeria syndrome: a premature aging disease. Molecular neurobiology. 2018;55(5):4417–4427. 10.1007/s12035-017-0610-7 28660486

[36] Putnam NC. Searching MEDLINE free on the Internet using the National Library of Medicine’s PubMed. Clinical excellence for nurse practitioners: the international journal of NPACE. 1998;2(5):314–316. 10455581

[37] Goodfellow I, Bengio Y, Courville A. Deep Learning. MIT Press; 2016.

[38] Nurzynska K. Deep Learning as a Tool for Automatic Segmentation of Corneal Endothelium Images. Symmetry. 2018;10(60):1–16. 10.3390/sym10030060

[39] Chen C, Qin C, Qiu H, Tarroni G, Duan J, Bai W, et al Deep learning for cardiac image segmentation: A review. arXiv e-prints. 2019; p. arXiv:1911.03723. 10.3389/fcvm.2020.00025 PMC7066212 32195270

[40] LeCun Y, Bengio Y, Hinton G. Deep Learning. Nature. 2015;521(7553):436–444. 10.1038/nature14539 26017442

[41] Al-Kofahi Y, Zaltsman A, Graves Rea. A deep learning-based algorithm for 2-D cell segmentation in microscopy images. BMC Bioinformatics. 2018;19(365). 10.1186/s12859-018-2375-z 30285608 PMC6171227

[42] Erickson BJ, Korfiatis P, Akkus Z, Kline TL. Machine Learning for Medical Imaging. RadioGraphics. 2017;37:505–515. 10.1148/rg.2017160130 28212054 PMC5375621

[43] Huynh BQ G M Li H. Digital mammographic tumor classification using transfer learning from deep convolutional neural networks. JAMA. 2016;3(3, (034501)). 10.1117/1.JMI.3.3.034501 27610399 PMC4992049

[44] Krizhevsky A, Sutskever I, Hinton GE. ImageNet Classification with Deep Convolutional Neural Networks. In: Proceedings of the 25th International Conference on Neural Information Processing Systems—Volume 1. NIPS’12. Curran Associates Inc.; 2012. p. 1097–1105. Available from: http://dl.acm.org/citation.cfm?id=2999134.2999257.

[45] Zeiler MD, Fergus R. Visualizing and Understanding Convolutional Networks In: Fleet D, Pajdla T, Schiele B, Tuytelaars T, editors. Computer Vision—ECCV 2014. Lecture Notes in Computer Science. Springer International Publishing; 2014 p. 818–833.

[46] Simonyan K, Zisserman A. Very Deep Convolutional Networks for Large-Scale Image Recognition. arXiv:14091556 [cs]. 2014;.

[47] Russakovsky O, Deng J, Su H, Krause J, Satheesh S, Ma S, et al ImageNet Large Scale Visual Recognition Challenge. International Journal of Computer Vision (IJCV). 2015;115(3):211–252. 10.1007/s11263-015-0816-y

[48] Laga H, Guo Y, Tabia H, Fisher R, Bennamoun M. 3D Shape Analysis: Fundamentals, Theory, and Applications. United States: Wiley-Blackwell; 2019.

[49] Szegedy C, Toshev A, Erhan D. Deep Neural Networks for Object Detection. Advances in Neural Information. 2013;35:1915–1929.

[50] Girshick R, Donahue J, Darrell T, Malik J. Rich feature hierarchies for accurate object detection and semantic segmentation. In: Proceedings of the IEEE conference on computer vision and pattern recognition; 2014. p. 580–587.

[51] He K, Gkioxari G, Dollár P, Girshick R. Mask R-CNN; 2017. 10.1109/TPAMI.2018.2844175 29994331

[52] Dinggang Shen GW, Suk HI. Deep Learning in Medical Image Analysis. The Annual Review in Biomedical Engineering. 2017;19:221–248. 10.1146/annurev-bioeng-071516-044442 28301734 PMC5479722

[53] Carin L, Pencina MJ. On Deep Learning for Medical Image Analysis. JAMA. 2018;320(11):1192–1193. 10.1001/jama.2018.13316 30422287

[54] Srivastava N, Hinton GE, Krizhevsky A, Sutskever I, Salakhutdinov R. Dropout: a simple way to prevent neural networks from overfitting. The Journal of Machine Learning Research Archive. 2014;15(1):1929–1958.

[55] Giger M. Machine Learning in Medical Imaging. Journal of American College of Radiology. 2018;15(3PB):512–520. 10.1016/j.jacr.2017.12.028 29398494

[56] Badrinarayanan V, Kendall A, Cipolla R. SegNet: A Deep Convolutional Encoder-Decoder Architecture for Image Segmentation. IEEE Transactions on Pattern Analysis and Machine Intelligence. 2017;39(12):2481–2495. 10.1109/TPAMI.2016.2644615 28060704

[57] Wang W, Wang Y, Wu Y, Lin T, Li S, Chen B. Quantification of Full Left Ventricular Metrics via Deep Regression Learning With Contour-Guidance—IEEE Journals & Magazine. IEEE Access. 2019;7:47918–47928. 10.1109/ACCESS.2019.2907564

[58] Ronneberger O, Fischer P, Brox T. U-Net: Convolutional Networks for Biomedical Image Segmentation In: Navab N, Hornegger J, Wells WM, Frangi AFE, editors. Medical Image Computing and Computer-Assisted Intervention—MICCAI 2015. vol. 9350 of Lecture Notes in Computer Science. Springer International Publishing; 2015 p. 234–241.

[59] Cireşan DC, Giusti A, Gambardella LM, Schmidhuber J. Deep Neural Networks Segment Neuronal Membranes in Electron Microscopy Images. Nips. 2012;.

[60] Cireşan DC, Giusti A, Gambardella LM, Schmidhuber J. Mitosis detection in breast cancer histology images with deep neural networks. In: International Conference on Medical Image Computing and Computer-assisted Intervention (MICCAI). Springer; 2013. p. 411–418. 10.1007/978-3-642-40763-5_51 24579167

[61] Urakubo H, Bullmann T, Kubota Y, Oba S, Ishii S. UNI-EM: An Environment for Deep Neural Network-Based Automated Segmentation of Neuronal Electron Microscopic Images. bioRxiv. 2019; p. 607366. 10.1101/607366 31857624 PMC6923391

[62] Liu J, Li W, Xiao C, Hong B, Xie Q, Han H. Automatic Detection and Segmentation of Mitochondria from SEM Images using Deep Neural Network. In: 2018 40th Annual International Conference of the IEEE Engineering in Medicine and Biology Society (EMBC). IEEE; 2018. p. 628–631. 10.1109/EMBC.2018.8512393 30440475

[63] Dorkenwald S, Schubert PJ, Killinger MF, Urban G, Mikula S, Svara F, et al Automated synaptic connectivity inference for volume electron microscopy. Nature methods. 2017;14(4):435 10.1038/nmeth.4206 28250467

[64] Konishi K, Mimura M, Nonaka T, Sase I, Nishioka H, Suga M. Practical method of cell segmentation in electron microscope image stack using deep convolutional neural network. Microscopy. 2019;. 10.1093/jmicro/dfz016 31220299

[65] Caicedo JC, Roth J, Goodman A, Becker T, Karhohs KW, McQuin C, et al Evaulation of Deep Learning Strategies for Nucleus Segmentation in Fluorescence Images. IEEE Reviews in Biomedical Engineering. 2018;2:147–171. 10.1002/cyto.a.23863 PMC6771982 31313519

[66] Quan TM, Hildebrand DGC, Jeong W. FusionNet: A deep fully residual convolutional neural network for image segmentation in connectomics. CoRR. 2016;abs/1612.05360.

[67] Antropova N GM Huynh BQ. A deep feature fusion methodology for breast cancer diagnosis demonstrated on three imaging modality datasets. Med Phys. 2017;44(10):5162–5171. 10.1002/mp.12453 28681390 PMC5646225

[68] G V, P L, Coram M ea. Development and Validation of a Deep Learning Algorithm for Detection of Diabetic Retinopathy in Retinal Fundus Photographs. JAMA. 2016;316(22):2402–2410. 10.1001/jama.2016.17216 27898976

[69] Wang W, Wang Y, Wu Y, Lin T, Li S, Chen B. Quantification of full left ventricular metrics via deep regression learning with contour-guidance. IEEE Access. 2019;7:47918–47928. 10.1109/ACCESS.2019.2907564

[70] He K, Zhang X, Ren S, Sun J. Deep residual learning for image recognition. In: Proceedings of the IEEE conference on computer vision and pattern recognition; 2016. p. 770–778.

[71] Szegedy C, Ioffe S, Vanhoucke V, Alemi AA. Inception-v4, inception-resnet and the impact of residual connections on learning. In: Thirty-First AAAI Conference on Artificial Intelligence; 2017. p. 4278–4284.

[72] Agarwal T, Mittal H. Performance Comparison of Deep Neural Networks on Image Datasets. In: 2019 Twelfth International Conference on Contemporary Computing (IC3); 2019. p. 1–6.

[73] Bianco S, Cadene R, Celona L, Napoletano P. Benchmark analysis of representative deep neural network architectures. IEEE Access. 2018;6:64270–64277. 10.1109/ACCESS.2018.2877890

[74] Su D, Zhang H, Chen H, Yi J, Chen PY, Gao Y. Is Robustness the Cost of Accuracy?–A Comprehensive Study on the Robustness of 18 Deep Image Classification Models. In: Proceedings of the European Conference on Computer Vision (ECCV); 2018. p. 631–648.

[75] Karabağ C, Jones ML, Peddie CJ, Weston AE, Collinson LM, Reyes-Aldasoro CC. Segmentation and Modelling of the Nuclear Envelope of HeLa Cells Imaged with Serial Block Face Scanning Electron Microscopy. Journal of Imaging. 2019;5(9):75 10.3390/jimaging5090075 PMC8320948 34460669

[76] Zhang Z, Duan C, Lin T, Zhou S, Wang Y, Gao X. GVFOM: a novel external force for active contour based image segmentation. Information Sciences. 2020;506:1–18. 10.1016/j.ins.2019.08.003

[77] Karabağ C, Jones ML, Peddie CJ, Westor AE, Collinson LM, Reyes-Aldasoro CC. Segmentation And Modelling of Hela nuclear Envelope. In: 2019 IEEE 16th International Symposium on Biomedical Imaging (ISBI 2019); 2019. p. 1510–1513.

[78] Deerinck TJ, Bushong E, Thor A, Ellisman MH. NCMIR—National Center for Microscopy and Imaging Research. NCMIR methods for 3D EM: A new protocol for preparation of biological specimens for serial block-face SEM Microscopy; 2010.

[79] Canny J. A Computational Approach to Edge Detection. IEEE Transactions on Pattern Analysis and Machine Intelligence. 1986;8(6):679–698. 10.1109/TPAMI.1986.4767851 21869365

[80] Ebsim R, Naqvi J, Cootes TF. Automatic Detection of Wrist Fractures From Posteroanterior and Lateral Radiographs: A Deep Learning-Based Approach. In: International Workshop on Computational Methods and Clinical Applications in Musculoskeletal Imaging. Springer; 2018. p. 114–125.

[81] Szegedy C, Liu W, Jia Y, Sermanet P, Reed S, Anguelov D, et al. Going deeper with convolutions. In: Proceedings of the IEEE conference on computer vision and pattern recognition; 2015. p. 1–9.

[82] Ioffe S, Szegedy C. Batch Normalization: Accelerating Deep Network Training by Reducing Internal Covariate Shift. BT-Proceedings of the 32nd International Conference on Machine Learning, ICML 2015, Lille, France, 6-11 July 2015, (2015) 448–456; 2015.

[83] Szegedy C, Vanhoucke V, Ioffe S, Shlens J, Wojna Z. Rethinking the inception architecture for computer vision. arXiv preprint 2015. arXiv preprint arXiv:151200567. 2015;.

[84] Nguyen LD, Lin D, Lin Z, Cao J. Deep CNNs for microscopic image classification by exploiting transfer learning and feature concatenation. In: 2018 IEEE International Symposium on Circuits and Systems (ISCAS). IEEE; 2018. p. 1–5.

[85] Deng J, Dong W, Socher R, Li LJ, Li K, Fei-Fei L. Imagenet: A large-scale hierarchical image database. In: 2009 IEEE conference on computer vision and pattern recognition. IEEE; 2009. p. 248–255.

[86] Shelhamer E, Long J, Darrell T. Fully Convolutional Networks for Semantic Segmentation. IEEE Transactions on Pattern Analysis and Machine Intelligence. 2017;39(4):640–651. 10.1109/TPAMI.2016.2572683 27244717

[87] Falk T, Mai D, Bensch R, Çiçek Ö, Abdulkadir A, Marrakchi Y, et al U-Net: Deep Learning for Cell Counting, Detection, and Morphometry. Nature Methods. 2019;16(1):67 10.1038/s41592-018-0261-2 30559429

[88] Dong H, Yang G, Liu F, Mo Y, Guo Y. Automatic Brain Tumor Detection and Segmentation Using U-Net Based Fully Convolutional Networks In: Valdés Hernández M, González-Castro V, editors. Medical Image Understanding and Analysis. vol. 723 of Communications in Computer and Information Science. Springer International Publishing; 2017 p. 506–517.

[89] Karabağ C, Verhoeven J, Miller NR, Reyes-Aldasoro CC. Texture Segmentation: An Objective Comparison between Five Traditional Algorithms and a Deep-Learning U-Net Architecture. Applied Sciences. 2019;9(18):3900 10.3390/app9183900

[90] Kingma DP, Ba J. Adam: A Method for Stochastic Optimization. http://arxivorg/abs/14126980. 2014;.

[91] Murphy KP. Machine learning: a probabilistic perspective. MIT press; 2012.

[92] Jaccard P. Étude comparative de la distribution florale dans une portion des Alpes et des Jura. Bulletin del la Société Vaudoise des Sciences Naturelles. 1901;37:547–579. 10.5169/seals-266450

